# Harnessing Particle Ions for FLASH Radiotherapy

**DOI:** 10.3390/cells15191729

**Published:** 2026-09-22

**Authors:** Alex J. Bembridge, Jonathan R. Hughes, Jason L. Parsons

**Affiliations:** 1 Department of Cancer and Genomic Sciences, University of Birmingham, Edgbaston, Birmingham B15 2TT, UK; 2 School of Physics and Astronomy, University of Birmingham, Edgbaston, Birmingham B15 2TT, UK

**Keywords:** carbon ions, FLASH, helium ions, ionising radiation, proton beam therapy, radiotherapy

## Abstract

**Highlights:**

**What are the main findings?**

- FLASH radiotherapy remains an area of biological and clinical interest due to the normal tissue sparing effects, whilst also providing tumour control comparable to conventional dose rate radiotherapy.
- Delivery of FLASH radiotherapy using particle ions (protons, carbon and helium ions) has the further potential to maximise tissue sparing whilst also providing enhanced biological effectiveness in tumour cell killing.

**What are the implications of the main findings?**

- FLASH radiotherapy, particularly using particle ions, continues to hold promise in providing more effective treatment for cancer patients whilst minimising adverse side effects compared to photon radiotherapy using conventional dose rates.
- Continued research is needed to further explore the biological effects of FLASH using different particle ions to accelerate its clinical potential.

**Abstract:**

FLASH radiotherapy continues to be an emerging clinical treatment that utilises ultra-high dose rate radiation (>40 Gy/s) to elicit normal tissue sparing compared to conventional (CONV) dose rate delivery. This is termed the FLASH effect, which has also been demonstrated to lead to effective tumour control. FLASH sparing has been demonstrated in many studies using electrons and now increasingly with particle ions, particularly protons. Due to unique properties such as deeper penetrance and a narrow range of energy deposition providing more precision targeting, this makes hadron radiotherapy using FLASH an exciting innovation for cancer treatment to help improve outcomes for cancer patients. Furthermore, through the use of higher linear energy transfer (LET) particles such as helium and carbon ions, FLASH offers the potential to combine sparing of normal tissues with enhanced biological effectiveness in tumour cell killing, important for tumours that are more resistant to conventional photon radiotherapy. However, the physical and biological parameters by which the FLASH sparing effect occurs is complex and has not been fully characterised. Factors including total dose, pulse structure (dose and duration), beam energy and LET have all been reported to impact tissue sparing. Additionally, the underlying biological mechanism of action of FLASH remains a point of contention within the field. In this review, we provide an up-to-date report on FLASH studies conducted with particle ion therapies, including in vitro and in vivo studies, using different experimental models. We present both the cellular and molecular outcomes of the studies performed using protons, helium and carbon ions, plus other ion sources, and the accumulating evidence that demonstrate the strong clinical potential for these modalities in cancer treatment.

## 1. Introduction

The World Health Organization (WHO) estimated that in 2022, almost 10 million global deaths were caused by cancer [1]. Around half of all patients diagnosed with cancer are expected to have some form of radiotherapy during their disease [2]. Whilst radiotherapy acts as a powerful tool to destroy tumour tissue, normal tissue surrounding the tumour is often also damaged. This can lead to severe toxicity, along with acute and chronic adverse side effects, particularly when vital organs are proximal to the irradiation site. Additionally, some tumours are extremely resistant to treatment, requiring higher radiotherapy doses or radiotherapy–drug combinations that can cause further patient complications. For this reason, there has been a significant effort to optimise current radiotherapy techniques and develop new technologies to increase the therapeutic ratio of tumour control to normal tissue damage. One such advancement is the delivery of ionising radiation at ultra-high dose rates (UHDRs; >40 Gy/s), commonly referred to as FLASH radiotherapy [3]. Whilst the differential impact of using UHDRs was first suggested as far back as the 1960s and 70s [4,5,6], a seminal paper published in 2014 by Favaudon et al. renewed interest in the field [7]. In this publication, UHDR electrons were used to irradiate a mouse lung model and found that this spared normal tissue compared to CONV dose rate delivery without compromising tumour control. This phenomenon is known as the ‘FLASH effect’ and over the past decade there has been an enormous influx of FLASH-related research, particularly using in vivo models. Normal tissue sparing following UHDR delivery of electrons has been observed in whole brain mouse, mini-pig skin, cat patient and canine skin models [8,9,10]. In addition to requiring an average dose rate of >40 Gy/s, a minimum dose is also needed to produce a FLASH effect and, whilst this is still a point of discussion, a dose of at least 8 Gy has been suggested, with this being dependent on various additional factors such as experimental model of interest and physical beam parameters (average dose rate, instantaneous dose rate, dose per pulse, pulse width, pulse frequency, number of pulses/spills, scanning pattern, total irradiation time) [11]. Therefore, these additional parameters should be considered alongside the average dose rate when comparing and interpreting different studies. Whilst the reduction in normal tissue toxicity is paramount, FLASH radiotherapy also has the potential to improve tumour targeting by limiting the impact of organ motion as well as reducing patient treatment times.

The availability of electron beams that can deliver radiation at UHDRs has enabled important initial investigations. However, the low tissue penetrance of electrons means that electron FLASH radiotherapy is limited in its clinical potential to treat deep-seated tumours, albeit there is ongoing research into very-high-energy electrons (VHEE) which offer superior penetration compared to low-energy electrons [12]. In contrast, particle beam therapy, such as using protons and other heavy ions, provides a much more favourable dose–deposition profile with a narrow Bragg peak (Figure 1). This peak in energy deposition can be spread out by superimposing several Bragg peaks with varied initial beam energies to produce a spread-out Bragg peak (SOBP) that allows the whole tumour to be targeted whilst maintaining limited off-target toxicities. Combining the favourable characteristics of particle ions with the normal tissue sparing benefits of FLASH offers greater clinical potential for an improved radiotherapy treatment for cancer patients. With that said, facilities providing charged particle therapy and associated research infrastructure are both relatively rare and expensive. In recent years, more cyclotron and synchrotron accelerator facilities have been developed around the world to a point where there are currently 130 proton and 17 carbon ion therapy centres across the globe (Particle Therapy Co-Operative Group [13]). The development of these facilities has been accompanied by numerous UHDR irradiation studies using both in vitro and in vivo preclinical models but also human clinical trials to determine the feasibility of delivering these particles at UHDRs in cancer patients.

Whilst many in vivo studies have demonstrated a FLASH effect, the exact mechanism by which irradiation with FLASH dose rates controls tumours yet spares normal tissue remains unclear. The oxygen depletion hypothesis builds on the foundation that ionising radiation causes the radiolysis of water molecules in critical structures and the resultant free radicals, which are normally reversible, will react with oxygen molecules to produce irreversible free radicals that cause permanent damage [15]. UHDR radiation delivery is thought to result in a transient state of hypoxia which prevents any further damage from occurring and, as tumour tissue is often already hypoxic, this causes a differential effect between normal and tumour tissue [16]. However, the extent to which oxygen depletion alone can account for FLASH sparing is debated, and it has been suggested that whilst oxygen is depleted following FLASH irradiation, the levels of depletion are insufficient to induce a biologically beneficial state of hypoxia [17,18]. Nevertheless, a recent review has proposed a more integrated model in which partial oxygen depletion may still contribute to FLASH sparing through interactions with radiation-induced radical chemistry, namely through an alternative restitution process mediated by radiation-induced aqueous electrons [19]. Many other mechanisms have been proposed to explain the FLASH effect phenomenon, and it is likely that these are not independent of one another but related in terms of the sequence in which they occur post irradiation, for example, radical–radical recombination [20] and reduced DNA damage in normal tissues [21,22,23,24] but also less efficient repair of DNA damage in the tumour [25]. Other potential impacts on mitochondrial function, cell death pathways, immune response, stem cells, and blood vessels [26,27,28] have also been reported.

Here in this review, we discuss the FLASH studies that have utilised charged particle irradiations in a range of in vitro and in vivo models centred on improving the mechanistic understanding of the FLASH phenomenon. We also cover reports analysing the feasibility of using densely ionising particles, such as carbon and helium ions, that have significantly improved relative biological effectiveness (RBE) and therefore tumour control. These investigations could underpin an effective translation to the clinic and improve the safety and outcomes of radiotherapy treatment.

## 2. Proton Beam Therapy

Protons, as with all particle ions, deliver the radiation dose in a well-defined range called the Bragg peak. Protons are generally classified as low-LET radiation, although comparable to photon radiotherapy, there are moderate increases in LET at and around the Bragg peak [29]. The majority of FLASH studies using particle ions to date have been conducted using protons, which will be detailed below relative to in vitro and in vivo systems, as well as early-phase human clinical trials.

### 2.1. In Vitro Studies

Reports of in vitro proton FLASH have been particularly sparse, mainly because animal models have been used to investigate potential FLASH sparing effects in a more translationally relevant setting (Table 1). However, in vitro studies still act as vital avenues in uncovering the radiobiological mechanisms that result in the FLASH effect. A study using normal lung fibroblast cells performed irradiations with 4.5 MeV protons (LET = 10 keV/μm) at either 0.05 (identified as CONV dose rate delivery), 100, or 1000 Gy/s and subsequently measured endpoints such as clonogenic cell survival, γH2AX foci formation, induction of senescence, and the induction of inflammation [30]. No FLASH sparing was observed in terms of clonogenic survival at the dose rates investigated. However, there was a significant reduction in γH2AX foci formation when doses of 20 Gy were delivered at a dose rate of 1000 Gy/s compared to the lower dose rates (100 Gy/s and 0.05 Gy/s). As γH2AX is an indirect marker of DNA double strand break (DSB) signalling rather than solely a direct measure of DNA DSBs, these findings may alternately reflect altered signalling, chromatin responses or DNA repair kinetics. At this radiation dose, it is important to note that saturation of foci formation can occur, so quantification of these experiments may be difficult to perform and interpret. Interestingly, when compared to a CONV dose rate, 100 and 1000 Gy/s caused a reduction in the number of senescent cells one month after 20 Gy proton delivery, as quantified by percentage of β-galactosidase positive cells. Additionally, 24 h post irradiation, the fold change of the pro-inflammatory marker, TGFβ, compared to non-irradiated samples, was ~6.5-fold higher following a 0.2 Gy/s dose rate, but only 1.8-fold higher following a dose rate of 1000 Gy/s. This effect persisted for a month later, suggesting that FLASH dose rates can significantly affect both short- and long-term biological responses to ionising radiation. A more recent study has investigated the FLASH effect in both non-tumour (V79) and tumour (A549 and U251) cells in either normoxic or hypoxic (<2%) conditions but observed no FLASH sparing [31]. As with the survival studies previously mentioned, no significant differences in clonogenic cell survival were observed when any of these cell types were irradiated with 0–18 Gy doses of protons at FLASH versus CONV dose rates (>40 Gy/s versus 0.1 Gy/s) in either normoxic or hypoxic conditions. However, conversely, another study revealed that proton irradiations at a dose of 15 Gy and a dose rate of 100 Gy/s versus 0.33 Gy/s resulted in a FLASH sparing effect in lung fibroblast cells when using a cell viability assay instead of a clonogenic survival assay [32]. In any case, these two studies highlight the inconsistent results achieved using in vitro models with UHDR radiations regarding difference in cell survival.

For this reason, more recent FLASH studies have focused on more robust and complex in vitro models. For example, the first study with primary 3D lung organoids to investigate proton FLASH was performed at the Holland Proton Therapy Center [33]. However, following irradiation with dose rates of either 40 Gy/s (FLASH) or 0.25 Gy/s (CONV), no sparing effect for FLASH was seen when comparing both survival and DNA damage response endpoints, assessed by organoid formation assays and γH2AX staining, respectively. It is possible that an even more complex model with an immune component might be necessary to observe sparing, but also, it should be noted that this study used relatively low doses of 2 Gy and 8 Gy, which may not be high enough to induce FLASH sparing. A minimum dose for FLASH sparing was suggested in another study using purified supercoiled pBR322 DNA plasmids to directly assess radiation-induced DNA breakage following proton irradiation, where it was deemed that doses ≤10 Gy were insufficient to result in differences in DNA breakage [34].

### 2.2. In Vivo Studies

#### 2.2.1. Mice

Many of the in vivo proton studies investigating a FLASH effect have been performed in mice. Some of the first observations of a FLASH effect with protons were reported after abdominal irradiations of C57BL/6J mice [35]. Here, an UHDR (78 ± 9 Gy/s) of 15 Gy protons resulted in a FLASH sparing effect on normal tissue, indicated by a reduction in levels of intestinal fibrosis compared to the levels that resulted from the same dose at a CONV dose rate (0.9 ± 0.08 Gy/s). Importantly, pancreatic flank tumours in the same mice were irradiated and no sparing effect of the FLASH modality was observed. These findings were corroborated by another study using a murine normal skin toxicity model irradiated under either CONV (0.35–0.40 Gy/s) or FLASH (65–92 Gy/s) dose rates [36]. However, a limitation of this study was the lack of a tumour tissue comparison to ensure the sparing was specific to normal tissue. Despite this, in a follow-up study skin toxicity was measured using a C3H mouse mammary carcinoma model, and as with the previous findings, it was demonstrated that FLASH sparing did not extend to the tumour [37]. It has also been suggested that FLASH dose rates can actually improve tumour control, as demonstrated in an orthotopic Lewis Lung Carcinoma (LLC) mouse model in which a dose of ~18 Gy, delivered at 61.9 Gy/s, reduced the tumour burden and number of proliferating tumour cells when compared to a CONV dose rate of 0.9 Gy/s [38]. Comparatively, an alternative study irradiated mice with 18.5 Gy to the whole pelvis and whilst there was higher survival following FLASH (50–80 Gy/s) compared to CONV dose rates (0.6–1 Gy/s), there were no significant differences in the control of flank tumours [39]. More recent studies have been performed to monitor the effects of FLASH irradiation on the lungs and skin of a preclinical mouse model where FLASH sparing was observed at a higher dose rate of 500 Gy/s, compared to a 2 Gy/s dose rate [40]. Here, the sparing effect was attributed to reductions in both oxidative stress (as visualised through Nrf2 activation, 8-oxoguanine levels, and MPO expression) and inflammation (decreased serum IL-6, TNF-α, and IFN-γ). FLASH sparing of skin and soft tissue was also observed in a separate study that performed proton irradiations via proton beam scanning (PBS). Here, 35 Gy doses were delivered with average dose rates of 57.4 Gy/s (FLASH) or 1.0 Gy/s (CONV) to a 25 mm × 23 mm field, composed of 30 spots, on the mouse leg. The spatial and temporal parameters required to produce FLASH sparing are still being characterised, but these findings suggest that sparing can still be produced using different delivery systems such as PBS [41].

Aside from lung and skin models, whole-brain proton irradiations of mice have been performed [42]. Here, two separate dose rates of 60 Gy/s and 95 Gy/s were used to irradiate adult male Sprague Dawley rats with 18 Gy protons, and which were compared against a CONV dose rate of 1 Gy/s. Whilst a large range of cognitive tests revealed no dose rate dependence on toxicity, protein analyses did reveal slight differences. Compared to sham treatment, only irradiation with 95 Gy/s showed significant reductions in α-amino-3-hydroxy-5-methyl-4-isoxazolepropionic acid receptor (AMPA) in the hippocampus and dopamine receptor D1 (DRD1) and tyrosine hydroxylase (TH) in the neostriatum. This trio of proteins have an important role in learning, memory and motor behaviour, so the reason for no differences in cognitive functions between the dose rates is unclear. Interestingly, the effect of FLASH radiotherapy on bone structure has also been investigated. The hindlegs of juvenile C57BL/6 mice were treated with 11 Gy doses at 200 Gy/s, and they displayed reduced long-term bone marrow and muscular toxicities as demonstrated by preserved bone mineral density, bone marrow cellularity, and lower muscle fibrosis in FLASH versus CONV-irradiated mice [43]. These findings are consistent with a previous study that irradiated mice hindlegs with 15 Gy with a CONV proton dose rate, followed by hypofractionated reirradiation with 3 doses of 11 Gy at either FLASH or CONV dose rates [44]. Here, FLASH reduced the percentage of mice with tibial fractures from 83.3% to 20% compared to those re-irradiated with a CONV dose rate.

Whilst the investigations described above have used entrance-beam (low-LET) proton irradiations, normal tissue sparing following FLASH dose rates has also been observed when performing 250 MeV SOBP irradiation of mice hindlegs, suggesting that the FLASH effect is independent of proton LET [45]. Irradiations were delivered via PBS to the foot of each mouse with dose rates of either 0.35 Gy/s (CONV) or 60 Gy/s (FLASH). A similar study also irradiated mice hindlegs with protons via PBS but did so in the entrance plateau region only rather than at the SOBP [36]. Here, a FLASH sparing effect of acute toxicity was observed by comparing levels of skin damage and identifying that a 44–58% higher dose was needed to obtain the same biological effect following average field dose rates of 80 Gy/s (FLASH) versus 0.37 Gy/s (CONV). This compares similarly to the study using SOBP protons described above, where a 35–46% higher dose would be needed to observe the same toxicity in the FLASH compared to CONV despite the higher LET. Nevertheless, the retention of FLASH sparing is encouraging in terms of clinical translation given that predominantly SOBP irradiations are used to target whole tumours whilst limiting normal tissue damage.

As mentioned above, whilst oxidative stress and inflammation have been identified as factors contributing to normal tissue sparing effects of FLASH, in general, studies investigating the mechanisms are still sparse. A GBM patient-derived xenograft model has been utilised where tumour-bearing mice were irradiated with 10 Gy protons with either a CONV or FLASH dose rate (values not specified). This revealed a higher number of γH2AX foci-positive cells in the CONV-irradiated brain samples compared to the FLASH-irradiated samples, suggesting differences in DSB signalling or repair [46]. However, based on RNA sequencing and immunofluorescence data, it has been suggested that FLASH sparing may be a result of reduced apoptosis and a reduced depletion of stem cell population, in addition to upregulation of tissue and vascular repair pathways [47]. Furthermore, this study demonstrated that FLASH dose rates also reduced inflammation in mouse skin, bone and soft tissue following doses of 30 Gy or 45 Gy. Additionally, mouse brain models have been used to demonstrate lower levels of neuroinflammation through a reduction in CD68-positive cells and HMGB1 nuclear release following FLASH dose rates [48]. Collectively, these findings indicate that mechanisms including the DNA damage response, immune response, and cell death may all contribute to FLASH sparing. It is therefore becoming increasingly clear that the FLASH effect could be the sum of several biological response mechanisms, and this may explain why the FLASH effect is more consistent in complex in vivo models compared to in vitro experiments.

#### 2.2.2. Zebrafish

Zebrafish embryo models have been used for FLASH studies, particularly as these allow for high throughput of irradiation experiments. By using embryonic survival, spinal curvature and pericardial oedema as endpoints, it was reported that 10–45 Gy protons with a mean FLASH dose rate of 100 Gy/s predominantly had no significant impact on these measurements when compared to CONV dose rates of 0.08 Gy/s [49]. An exception was at a single dose point (23 Gy) where oedema rate was significantly reduced following FLASH 3 and 4 days post irradiation, possibly due to a minimum dose being required to elicit FLASH sparing. Nevertheless, the lack of FLASH sparing effects possibly relates to the lack of oxygen control. Indeed, a follow-up study that reduced the oxygen tensions for embryo incubations to <10 mmHg showed that this led to dose rate-dependent FLASH sparing through a reduction in radiation damage via differences in mean body length and malformation rates following irradiation with ~30 Gy protons at 300 Gy/s versus ~0.15 Gy/s [50]. However, the role of oxygen in the mechanism of FLASH remains a point of discussion as other studies using phosphorescence quenching methods and water phantom models have demonstrated that the amount of oxygen depleted following UHDR radiation is insufficient to produce a radioprotective hypoxic state in bulk tissue [17,18].

A more recent zebrafish study has compared the impact of using entrance dose protons (0.4 keV/µm), SOBP protons (2.5 keV/µm), or electrons (0.27 keV/µm) at FLASH dose rates of 240, 600 and 0.9 × 10^5^ Gy/s, respectively [51]. Corresponding reference CONV dose rates of 0.33 (entrance dose protons), 0.5 (SOBP protons) and 0.1 Gy/s (electrons) were also delivered, and a wide dose range of 15–95 Gy was used for all three dose rate modalities. When measuring spine curvature and embryonic length, dose effect curves for the SOBP proton beam indicated lower doses were required to produce the same biological effect, demonstrating the increased RBE that can be achieved. Significant FLASH sparing was observed for all three beams, but this was dependent on the biological endpoint. For example, FLASH sparing was seen for curved spine and eye diameter for the entrance dose protons, whereas curved spine and embryo length displayed sparing for the SOBP protons. As for electrons, all four measured endpoints (pericardial oedema, curved spine, embryo length, and eye diameter) showed FLASH sparing following UHDR delivery compared to the CONV dose rate. The sparing effect was more pronounced for all four endpoints when irradiations were performed with the electron beam, possibly due to a much greater dose rate being used. It would be interesting if the study could have been performed using equivalent dose rates relative to particle type and LET. Another study also used four biological endpoints (viability, length, spine curvature, and pericardial oedema) following irradiation with FLASH versus CONV dose rate protons (8–9 kGy/s or 0.2 Gy/s, respectively) to deliver overall doses of 30 or 40 Gy [52]. FLASH sparing was observed for embryo length at 30 and 40 Gy, as well as for pericardial oedema (only at 30 Gy). Despite the evidence described above, variabilities in the findings and the sensitivity of the individual biological endpoints remain a problem. For example, the dose response curve for pericardial oedema was shown to become saturated at 30 Gy and beyond [51], whereas with embryo length, plateauing did not occur until at least 60 Gy doses were reached.

In other most recent studies, a FLASH sparing effect was again observed, but it was dependent on the endpoint being measured and the oxygenation status. FLASH sparing for pericardial oedema in normoxic conditions was seen following an expected dose of 30 Gy, although the FLASH dose rate of 317 Gy/s also resulted in greater development of eye and head size in hypoxic conditions compared to the CONV dose rate of 0.6 Gy/s [53], suggesting the tissue microenvironment as a key factor in mediating the FLASH response. To provide more mechanistic detail, a study using zebrafish embryos irradiated using a 68 MeV cyclotron with 30 Gy protons at a dose rate of either 0.18 Gy/s (CONV) or >5100 Gy/s (FLASH) revealed changes in DNA single strand break formation after 2 h, as directly measured using an alkaline comet assay [54]. However, no significant FLASH sparing for viability, spinal curvature, or body length was identified, and it is notable that oxygen tensions at the time of irradiation were not controlled. Selected gene expression analysis revealed that cell cycle regulation was the most differentially affected process 6 h post irradiation, whereas immune response, apoptosis, and p53 regulation showed the greatest difference 24 h post irradiation. Specifically, *cdkn1a*, an inhibitor of cell cycle progression in G1, was 1.25-fold higher in CONV versusFLASH groups 6 h post irradiation, and *mdm2*, a ubiquitin ligase responsible for p53 degradation, was 1.22-fold higher after CONV irradiation 24 h post irradiation. If FLASH dose rates can reduce the degradation of p53, this would allow it to induce the transcription of many DNA damage response genes, including *cdkn1a*, which promotes cell cycle arrest and allows for DNA damage repair to improve overall cell survival. Following this, quantification of the apoptotic marker, cleaved caspase 3 (cC3) and proliferation marker, phospho-histone 3 (pH3) analysed in embryonic cells from the tail and head region at 6 and 20 h post irradiation, revealed that the only significant difference was in the tail 20 h post irradiation, where FLASH dose rates reduced overall apoptosis levels. No significant changes in proliferation were observed across any of the conditions. Immune response genes were also found to be differentially expressed between dose rates, although no significant differences in neutrophil counts 2 h post irradiation in the head or tail were observed. It could be argued that a single timepoint may be insufficient to capture differences in immune response between dose rates, and that a longer time course profile is needed to capture the short and long-term effects of FLASH.

#### 2.2.3. Chick Embryos

The chick embryo chorioallantoic membrane (CAM) model has become an enticing avenue to investigate the radiobiology of FLASH at an in vivo preclinical level, as its partially immune-deficient properties allow for implantation of tumour cells, organoids and organotypic tissue slice cultures without the associated immune response [55]. This model also provides a more representative embryogenesis model to that of a human compared to zebrafish. In one study, fertilised eggs of the Japanese quail (*C. coturnix japonica*) were irradiated on the 5th–6th day of incubation with either CONV (<1 Gy/s), FLASH (~100 Gy/s), or SPLASH (single-pulse FLASH, ~10^5^ Gy/s) dose rates [56] at different doses relative to the Bragg peak (SOBP or plateau). Most embryos and chicks died or had significant deformation following irradiation within the plateau region of the Bragg curve, probably due to the higher doses being delivered. Embryos irradiated with 8.5 Gy displayed an increase in micronuclei-positive erythrocytes when a CONV dose rate was used, as opposed to either FLASH or SPLASH. Whilst this increase was significant, the difference in micronuclei-positive cells was only ~1% between CONV and FLASH. In contrast, significant increases in both body length and weight were observed following FLASH compared to CONV, indicating developmental FLASH sparing. Interestingly, when comparing FLASH to SPLASH, no difference in micronuclei was observed, whereas an even greater sparing effect on both body length and weight of the embryos was seen. In contrast to the embryo experiments, no significant differences in cytogenetic abnormalities (binucleate erythrocytes, anucleate erythrocytes, and micronuclei) were observed in the hatched chicks between irradiation modes. This could be due to a lower radiation dose (4 Gy) being used. Finally, chicks irradiated in the CONV mode appeared to be significantly less mobile compared to FLASH and SPLASH modes, indicating potential FLASH sparing of cognitive function. Unfortunately, no tumour tissue comparisons were investigated in this study, which is possible as demonstrated in a separate electron irradiation study [57].

### 2.3. Clinical Trials

The feasibility of a FLASH proton delivery system as a form of radiotherapy treatment has been assessed in a world first clinical trial (FAST-01), where 10 patients with bone metastases in the extremities were treated with 8 Gy protons at a dose rate of 51–60 Gy/s [58]. This form of treatment was shown to be clinically feasible; plus, the safety and efficacy (pain relief) data was consistent with that of conventional forms of radiotherapy delivery. Whilst positive, this study had some limitations related to the small population size, the number of anatomic sites that were irradiated and assessed, along with no CONV dose rate comparison. A subsequent clinical trial (FAST-02) is currently being undertaken to assess the efficacy and safety of proton FLASH therapy treatment in 10 patients with bone metastases in the thorax [59]. Assuming pain response and adverse effects show similar results to the FAST-01 study, the additional data in different anatomical sites will support an effective clinical transition of proton FLASH radiotherapy treatment.

**Table 1:** Summary of FLASH versus CONV studies using proton beam therapy.

Model	Ion Source	Beam Energy (MeV)	LET (keV/μm)	Dose (Gy)	FLASH Dose Rate (Gy/s)	CONV Dose Rate (Gy/s)	Outcome	Reference
Normal human lung fibroblasts (IMR90)	Proton	4.5	10	0–20	100 or 1000	0.05	No influence on cell survival FLASH dose rates reduced senescence and TGFβ1 levels	[30]
Chinese hamster lung fibroblasts (V79), human brain glioblastoma (U-251), and human lung adenocarcinoma (A549) cells	Proton	230	N/A	0–18	50 or 65	0.1	No significant difference in survival of tumour or non-tumour cells under normoxic or hypoxic irradiation conditions	[31]
Primary bronchial epithelial cell (PBEC) organoids (COPD patients)	Proton	250	N/A	2 and 8	40	0.25	No significant changes in DNA damage or gene expression between dose rates Detrimental effect on FLASH-irradiated organoid formation	[33]
pBR322 plasmids	Proton (entrance, BP, and SOBP)	250	N/A	2–50	400 (entrance) 1300–1400 (BP) 500 (SOBP)	1	Plasmid DNA strand breakage independent of dose rate at doses below 10 Gy	[34]
C57BL/6J mice (intestine), flank tumours derived from KPC autochthonous PanCa model	Proton	230	N/A	12–18	78 ± 9	0.9 ± 0.08	FLASH reduced loss of proliferating cells in intestinal crypts and reduced intestinal fibrosis Equivalent tumour control across dose rates	[35]
CDF1 mice (skin)	Proton	244 (CONV), 250 (FLASH)	N/A	23.2–53.5	0.35–0.73	65–92	FLASH normal tissue sparing of acute skin damage	[36]
CDF1 mice (skin and C3H mouse mammary carcinoma)	Proton	244 (CONV), 250 (FLASH)	N/A	40–60	71–89	0.33–0.63	Equivalent tumour control Normal tissue FLASH sparing—acute skin damage and radiation induced fibrosis	[37]
CDF1 mice (skin)	Proton (SOBP)	244 (CONV), 250 (FLASH)	N/A	19.9–65.9	60	0.34	Normal tissue FLASH sparing—acute skin damage and radiation induced fibrosis	[45]
C57BL/6J mice (skin)	Proton	250	N/A	15 or 35	57 or 115	1	FLASH dose rates reduced plasma and skin levels of TGF-β1, skin toxicity and leg contracture Equivalent control of MOC1 and MOC2 carcinoma by FLASH and CONV	[41]
C57BL/6 and C3H/HeJ mice—fibrosarcoma syngeneic tumours	Proton	230	N/A	30 or 45	69–124	0.39–0.65	FLASH reduced severe morbidity and radiation-induced skin damage FLASH mitigated inflammatory response and reduced muscle and bone damage Equipotent tumour control in FLASH or CONV Reduced TGFβ1 induction following CONV dose rates upregulated apoptosis, keratin signalling and keratinocyte differentiation	[47]
C57BL/6 mice -Orthotopic Lewis Lung Carcinoma (LLC) tumour model	Proton	244 (CONV), 250 (FLASH)	N/A	18	60	1	FLASH more efficiently eliminated tumour cells FLASH increased cytotoxic CD8^+^ T cell infiltration FLASH decreased number of immunosuppressive Tregs FLASH decreased number of PD1/PD-L1 markers in lung	[38]
C57BL/6 mice—lung and skin	Proton	230	N/A	60	500	2	FLASH decreased pneumonitis and fibrosis FLASH reduced levels of oxidative stress markers (Nrf2, 8-OHdG, MPO) and inflammatory markers (IL-6, TNF-α, and IFN-γ)	[40]
C57BL/6 mice—flank tumours, colonic tissue	Proton	48.7	N/A	18 or 18.5	50–80	0.6–1	FLASH improved overall survival Significantly higher cell proliferation in distal colon following FLASH FLASH resulted in lower cleaved caspase-3 staining Comparable tumour control across dose rates	[39]
C57BL/6 mice—brain	Proton (Bragg peak)	146.6	4.5–10.2	10	120	0.17	Significantly lower radiation-induced fibrosis (RIF)-positive cells following FLASH FLASH preserved microvasculature and reduced HMGB1-mediated inflammation	[48]
C57BL/6 mice—bone	Proton (SOBP)	142.4	2.1	11 (FLASH) and 16.64 (0.64 × 26 spills (CONV)	200	0.2	FLASH preserved bone microarchitecture, bone marrow cellularity, and reduced muscle fibrosis	[43]
C57BL/6 mice—intestine, skin, and bone	Proton	230	N/A	12 + 12 or 12 + (3 × 6.4) (intestine) 15 + (3 × 11) (skin and bone)	127.39 ± 5.95 (intestine) 106.5 ± 3.8 (skin and bone)	0.79 ± 0.04 (intestine) 0.7 ± 0.07 (skin and bone)	FLASH induced less intestinal fibrosis and increased survival FLASH reduced lymphedema and fibrosis in skin FLASH reduced frequency of tibial fractures	[44]
GBM PDX mice (XD456)	Proton	250	N/A	10	Not stated	Not stated	FLASH-irradiated mice demonstrated reduced number of γH2AX positive cells No significant difference in DNA/RNA damage	[46]
Sprague Dawley rats—brain	Proton (Bragg peak)	250 (FLASH) or 245 (CONV)	N/A	18	60 or 95	1	No differences in cognitive toxicity	[42]
Zebrafish embryo	Proton	224	N/A	10–40	100	~0.08	No influence of FLASH dose rates on embryonic survival or rate of spinal curvature FLASH reduced rate of pericardial edema (only at 23 Gy)	[49]
Zebrafish embryo	Proton	224	N/A	~30	~300	~0.15	Embryo survival and hatching rate not influenced by dose rate FLASH reduced pericardial edema and rates of spinal curvature	[50]
Zebrafish embryo	Proton	228	N/A	33.6 ± 1.8 (CONV) 33.9 ± 1.0 (FLASH)	317	0.6	FLASH reduced pericardial oedema in normoxic and mitigated effects on eye and head development in hypoxic conditions	[53]
Zebrafish embryo	Proton, Proton SOBP	225	0.4 (entrance) or 2.5 (SOBP)	20–95	240 (entrance) or 600 (SOBP)	1.65 (entrance) or 2.5 (SOBP)	FLASH sparing of spinal curvature rate FLASH sparing of eye diameter deficit (monoenergetic protons) FLASH sparing of embryo length (SOBP protons)	[51]
Zebrafish embryo	Proton	68	N/A	30 or 40	8200 to 9200	0.2 to 0.25	FLASH sparing of body length FLASH sparing of pericardial oedema at lower dose No differences in survival or spine curvature	[52]
Zebrafish embryo	Proton	68	N/A	30.4 ± 0.5 (CONV) or 30.5 ± 0.5 (FLASH)	5100–10,800	0.18 ± 0.026	Comparable viability and development between dose rates Reduced DNA damage following FLASH Dose-rate dependent cell cycle- and p53 regulation-related gene expression Higher apoptosis levels in tails following CONV but comparable neutrophil migration	[54]
Chick embryo	Proton (entrance and SOBP)	160	~0.03	2–14	~100 (FLASH) ~10^5^ (SPLASH)	<1	FLASH and SPLASH spared body weight, length, and micronuclei in embryos Chicks irradiated with CONV were less active than other groups	[56]

## 3. Carbon Ion Therapy

Compared to protons, the significant advantage of carbon ions is that they have reduced lateral scattering and a significantly higher LET which enables better dose conformation and increased RBE to the tumour [60]. Clinically delivered carbon ion therapy has an average LET in the range of 40–80 keV/µm, which produces an RBE of ~2–3 [61]. However, carbon ions display a nuclear fragmentation tail that can result in a low dose delivery beyond the Bragg peak and target volume [62]. Several in vitro and in vivo studies performing carbon ion FLASH irradiation have been reported (Table 2).

### 3.1. In Vitro Studies

One of the first carbon ion FLASH experiments was conducted using the Heidelberg Ion-Beam Therapy Center (HIT) synchrotron to irradiate Chinese Hamster Ovary (CHO)-K1 cells with 7.5 Gy carbon ions at a dose rate of either 70 Gy/s (FLASH) or 0.6 Gy/s (CONV) at various oxygen tensions (0–21% O_2_) [63]. A FLASH sparing effect was observed when cells were irradiated at oxygen tensions between 0.5 and 4%. This contrasts with another study using human lung fibroblast, HFL1, and human salivary gland tumour, HSGc-C5, cell lines to enable comparisons between normal and tumour cell responses to be made. Here, no FLASH sparing was observed following 1–3 Gy irradiations with carbon ions at a dose rate of 96–195 Gy/s compared to 8–13 Gy/s [64]. It is notable that in the previous study mentioned, samples were irradiated viaPBS, which can increase the variability in dose rate, whereas the current study irradiated a single spot with much lower doses within a single spill duration that can also introduce variations and may explain the observed differences. Another study investigated the impact of carbon FLASH irradiation of human salivary gland cells (HSGc-c5), human dermal fibroblasts (HDFs) and human lung bronchial epithelial cells (Nuli-1), where they observed FLASH sparing, suggested via a reduction in cell death following carbon ion irradiation delivered with a dose rate of 100 Gy/s compared to a CONV dose rate of 1.6 Gy/s [65]. Interestingly, the cell sparing effect was more readily observed when a higher LET was employed, and survival curves from this study also indicate that doses higher than 3 Gy were required to see a sparing effect. From a mechanistic perspective, a reduction in γH2AX foci, suggesting altered DSB-associated signalling and/or repair kinetics was observed following UHDR delivery of carbon ions. In another study in which B16F10 murine melanoma and Pan02 murine pancreatic adenocarcinoma cell lines were irradiated with carbon ions at either 1.16 Gy/s (CONV) or 380 Gy/s (FLASH), the impact on endoplasmic reticulum (ER) stress was measured [66]. Here it was shown that UHDR carbon ions may result in an enhanced immune response with a reduction in the immunosuppressive protein and programmed cell death 1 ligand (PD-L1) but increases in immune-promoting proteins, calreticulin (CRT) and major histocompatibility complex class (MHC)-I. However, these findings were not consistent across both cell lines, and further evidence would be necessary to confirm the role of the immune response in the FLASH effect.

### 3.2. In Vivo Studies

The first in vivo carbon ion FLASH study used an osteosarcoma mouse model which generates metastases in the lung [67]. A FLASH sparing effect was observed through a reduction in muscle tissue damage and a more organised morphology when mice were irradiated with carbon ions at ~100 Gy/s versus ~18 Gy/min. Tumour volume was also significantly reduced following FLASH, but it was suggested this could be related to CONV irradiation causing oedema of the hind leg due to inflammation of the normal tissue surrounding the tumour. Strikingly though, FLASH dose rates caused a significant reduction in the amount of metastatic tissue present in the lung compared to both CONV and sham irradiated mice. To assess the impact of FLASH carbon ions through a more clinically relevant delivery, the osteosarcoma mouse model was irradiated with 20 Gy at the SOBP region where a higher LET can be employed (65–85 keV/μm) compared to irradiations within the plateau region of the Bragg curve [68]. FLASH sparing of normal tissue was evident through a reduction in radiation-induced fibrosis; however, in terms of tumour control, FLASH and CONV dose rates were equipotent and metastatic development was not affected by dose rate when irradiating within the Bragg peak region as it was with entrance dose irradiations. Nevertheless, more comparisons are needed to assess the role of LET in the response of carbon ion irradiations related to FLASH delivery.

FLASH irradiation has been shown to cause reductions in skin toxicity in many preclinical proton FLASH studies, and these have also been demonstrated with the use of carbon ions. FLASH sparing was observed in a study where C3H/He mice received doses of 34–58 Gy carbon ions to the hind limb at dose rates of either 0.047–0.051 Gy/s (CONV) or 166.7–190.8 Gy/s (FLASH) [69] and which showed that skin toxicity was clearly reduced following UHDR irradiation. The first investigation into the impact of FLASH carbon ion irradiation on the gastrointestinal tract has also been performed, with contrasting results to other carbon ion FLASH studies thus far [70]. Following administration of 12–18 Gy carbon ions to C3H/HeSlc mice at dose rates of either 0.3 Gy/s (CONV) or 96 Gy/s (FLASH), no FLASH sparing effect was observed regarding intestinal toxicity. It is possible that a higher dose would be required to observe a FLASH sparing effect in this model, but also the number of samples used for histological analysis in this case was low and the use of other endpoints may be required to provide supporting data.

## 4. Helium Ion Therapy

Like carbon ions, helium ions also display less lateral scattering, higher dose weighted LET values (~4 to ~40 keV/μm), and higher RBE values (~1.3 to ~3) compared to protons [71,72]. However, helium ions benefit from a reduced fragmentation tail meaning there is a more uniform LET profile and confined dose distribution. Limited availability in terms of helium ion facilities has stunted the progression in understanding helium ion irradiation effects since its feasibility for treating meningioma and uveal melanoma was demonstrated [73,74]. The development of facilities such as the Heidelberg Ion-beam Therapy Center (HIT) have allowed the first FLASH helium ion studies to be performed (Table 3). Experiments with two human lung epithelial cell lines (A549 and H1437) showed a FLASH sparing effect when irradiated with 8–12 Gy helium ions at a dose rate of 185 Gy/s versus 0.12 Gy/s [75]. The greater survival was also accompanied by a reduction in γH2AX foci, indicating a potentially altered DNA damage response following UHDR radiation.

To validate this finding in more complex organisms, the development of zebrafish embryos has been compared following helium ion irradiations at either 0.25 ± 0.02 Gy/s (CONV) or 9.7 ± 0.82 kGy/s (FLASH) [76]. UHDR helium ion irradiations resulted in zebrafish embryos of greater length (18 Gy and 24 Gy) and lower spine curvature ratios (24 Gy only) than CONV irradiations, indicative of FLASH sparing. A recent study has furthered this evidence by observing a FLASH effect through the use of two mice models to assess the impact of UHDR helium ions on both a normal tissue response and tumour control [77]. For the latter, a subcutaneous carcinoma tumour model was established via the injection of A549 adenocarcinoma cells into female NMRI mice and these were irradiated with 12.5 Gy at 0.2 Gy/s or 250 Gy/s. FLASH dose rates demonstrated equivalent tumour control to CONV dose rates, with some indications that animals irradiated with FLASH survived slightly longer than the CONV-irradiated group. To assess effects on normal tissue toxicity, specifically in the brain, the cortices of female C57BL/6 mice were irradiated with 10 Gy at either 0.2 Gy/s or 141 Gy/s. Differences in DSB-associated signalling and/or repair kinetics were observed through γH2AX foci, which was significantly lower when irradiating with FLASH at both 1 h and 7 days post irradiation, but also this resulted in reduced activation of microglia/macrophages (CD68^+^) and the preservation of endothelial blood–brain barrier structural integrity. Together, these findings highlight the benefit of FLASH helium ion radiotherapy in terms of neuroprotection. However, substantially more studies comparing normal and tumour responses following helium ion FLASH are needed to evolve these initial findings.

The extension of FLASH irradiation to higher-LET particles such as helium and carbon ions is interesting due to their increased RBE compared to protons and other low-LET radiation modalities, and their favourable dose-depth distribution providing inherent normal tissue sparing. However, the currently limited literature on helium and carbon ion FLASH, whilst illustrating feasibility, demonstrates mixed results depending on irradiation parameters (dose, dose rate, LET), biological model, and experimental endpoint. Indeed, increasing LET alters the complexity of DNA damage due do the differential radiation track structure and energy deposition, which changes the biological response and may therefore influence the mechanisms and conditions required for the FLASH effect at these higher LETs. Therefore, future systematic studies using defined models and experimental setups that are comparable should focus on the magnitude to which particle type and LET influences the FLASH effect and determine whether the advantages of higher-LET particle radiotherapy can be combined with FLASH sparing.

## 5. Other Ion Sources

Neon ions are of interest with FLASH delivery due to their very high LET and potential to overcome tumour radioresistance, and they are currently the subject of testing in the HI-FLASH project based at GSI (Darmstadt, Germany) [78]. This project will test neon ions at UHDRs in a syngeneic F98/Fischer rat GBM model and use neurotoxicity and survival as endpoints. Like neon, oxygen ions may be a more suitable option for radioresistant hypoxic tumours as it displays a higher dose averaged LET value when compared to carbon ions [79]. On the other hand, beryllium and boron beams show promise for smaller tumours closer to the surface as they deposit a low dose in the plateau region and a high dose within the target tumour volume [80]. Pairing the advantageous properties of these ions with FLASH could further expand their therapeutic potential. Of course, the limiting factor here is the infrastructure required for the generation of these beams and the accompanying cost.

Whilst biological research into FLASH radiotherapy has been expanding rapidly over the past decade, technological advancements have led to novel UHDR delivery systems such as laser-driven ion sources that have the capability of delivering extreme instantaneous dose rates (>10^9^ Gy/s) that are multiple orders of magnitude higher than those currently used for FLASH studies [81,82]. Current limitations of using laser-driven ion acceleration to induce a FLASH effect include challenges achieving the required average dose rate and total dose for FLASH (>40 Gy/s, >8 Gy), as well as irradiation of larger areas/volumes and deep-seated tumours. This is due to constraints on laser-pulse repetition rates, beam energies, field sizes, and dose uniformity and homogeneity. As a result, a number of studies that have investigated the radiobiological effect of laser-driven protons and carbon ions have been unable to show a significant FLASH sparing effect between UHDR and CONV dose rates, which could be due to the inability to satisfy the generally accepted criteria for the FLASH effect due to either multi-shot approaches or lower total doses [81,83,84,85,86,87,88,89,90,91,92,93]. In a recent study, radiation doses up to 10 Gy (0.7 Gy/shot, 1.5 × 10^8^ Gy/s peak dose rate) were used to investigate the radiosensitivity of radioresistant U87 and SF763 glioblastoma cell lines [94]. Compared to X-rays (200 kV, 15 mA) and conventionally accelerated protons (235 MeV cyclotron, degraded to 20 MeV), the efficiency of laser-driven protons (200 TW laser, TNSA) was similar in terms of cell killing and DSB induction. Despite the historical technical challenges associated with laser-driven ion sources for FLASH, a study showed they were able to achieve a dose rate of >10^9^ Gy/s in a single shot of 20 Gy over a 1 cm^2^ treatment area [82]. Exploratory proof-of-principle experiments showed that single radiation doses up to 10.8 Gy demonstrated similar anti-tumour effectiveness in U87 glioblastoma cells compared with data from the author’s previous study comparing laser-driven protons (0.7 Gy per shot) and conventionally accelerated protons [82,94]. Interestingly, when detecting levels of 8-oxoguanine, it was observed that normal MRC5 human fibroblast cells had a 5.7% lower oxidative burden than the U87 glioblastoma tumour cells, suggesting a normal/tumour cell differential response to FLASH [82]. This concurs with findings from another group that utilised the BELLA PW laser facility at the Lawrence Berkeley National Laboratory (LBNL) to irradiate normal human prostate epithelial cells (RWPE1) and prostate cancer cells (PC3) with 7 to 35 Gy protons at an instantaneous dose rate of 10^7^ Gy/s [95]. Here, normal cells displayed significantly greater survival compared to tumour cells for doses above 7 Gy. Finally, zebrafish embryos irradiated with 10 Gy 4 h post fertilisation suggested that there was a reduction of 25% in zebrafish length when compared to the non-irradiated control, although CONV dose rates were not used. It remains to be seen whether the extremely high dose rates achieved with lasers operate within the same parameters as FLASH or will result in a distinct biological response entirely, which is a focus of the LhARA (Laser-hybrid Accelerator for Radiobiological Applications) initiative [96].

## 6. Concluding Remarks and Future Directions

Preclinical evidence in general supports that delivering ionising radiation at UHDRs could improve radiotherapy treatment outcomes due to the ability to escalate the dose with a reduction in healthy tissue toxicity and without compromising tumour control. The discrepancy between in vitro and in vivo responses suggests that the FLASH effect may not be a purely cell-autonomous radiobiological phenomenon, and FLASH sparing may instead arise from the convergence of the interactions of multiple biological systems, cellular responses, and organism/tissue-level factors. These factors include the immune response, vascular effects and the tumour microenvironment, and they may help explain why the FLASH effect is more consistently observed in more complex in vivo models compared to simple in vitro systems. Nevertheless, the mechanism(s) behind the FLASH effect are still unclear, and understanding these are vital for enabling effective translation to the clinic. Both in vitro and in vivo studies will therefore have key roles in uncovering these mechanisms. Specifically, the impact of dose rate on processes such as oxygen depletion, the DNA damage response, and immune responses need further investigation both in vitro and in vivo. In vitro models such as cell monolayers, 3D spheroids, and patient-derived organoids offer easier avenues into investigating the impact of dose rate on survival and, more mechanistically, effects on DNA damage and the role of oxygen. Investigating the immune response is more challenging in vitro but, as highlighted in this review, many groups have utilised in vivo models, such as mice, to compare the extent of fibrosis as well as the infiltration of immune cells. Whilst some in vivo models, such as zebrafish embryos, have been useful in highlighting the sparing of healthy tissue with FLASH, ideally future in vivo studies will compare both healthy and tumour tissue response. This difference is the key advantage of FLASH radiotherapy and largely previous in vivo studies have focused solely on the healthy tissue sparing aspect of the response. Thus, models such as the CAM model and mice (xenograft or syngeneic) may offer better alternatives as comparisons of tumour versus healthy tissue response can be made directly within the same organism. However, it is important to not neglect the mechanistic basis of any phenotype seen, especially considering this aspect of FLASH radiotherapy is still poorly understood.

Studies up until this point suggest that dose rate is not the only factor influencing the FLASH effect, and other parameters such as total dose, pulse structure (dose and duration), and beam energy also contribute. In addition, whilst protons and heavier ions offer the potential to improve radiotherapy outcomes due to their narrow range of energy deposition, ability to treat deep-seated tumours, high LET properties (more so for heavy ions), and inherent normal tissue protection, further research is required to fully realise the potential of using particle ions for FLASH. To that end, several research priorities should be addressed to support the clinical implementation of particle ion FLASH. Firstly, further investigation is needed into how the FLASH effect is impacted by high-LET and particle-specific differences, comparing both entrance to Bragg peak (and SOBP) regions. Secondly, the mechanisms underlying the FLASH effect, and how these responses are modified by particle type or LET, require further investigation. Comprehensive reporting and greater standardisation of physical beam parameters and irradiation conditions will allow meaningful comparisons between studies. Ideally, these would use well-defined, clinically relevant models that allow for tumour and healthy tissue comparisons under physiological conditions to establish contributions of oxygen, DNA damage responses, immune effects and radiation chemistry. Finally, technical advancement of particle FLASH-capable systems is key to enabling end user accessibility and development of cost-effective and compact beam delivery and accelerator systems, ultimately facilitating wider patient access.

Pairing charged particles with FLASH could potentially enable a larger range of recurrent and radioresistant tumours to be treated with greater biological effectiveness, whilst mitigating healthy tissue toxicity further. Provided that the technical and biological challenges can be addressed, this approach has the potential to improve therapeutic efficacy in clinical treatments and increase patient quality of life.

## Figures and Tables

**Figure 1:**
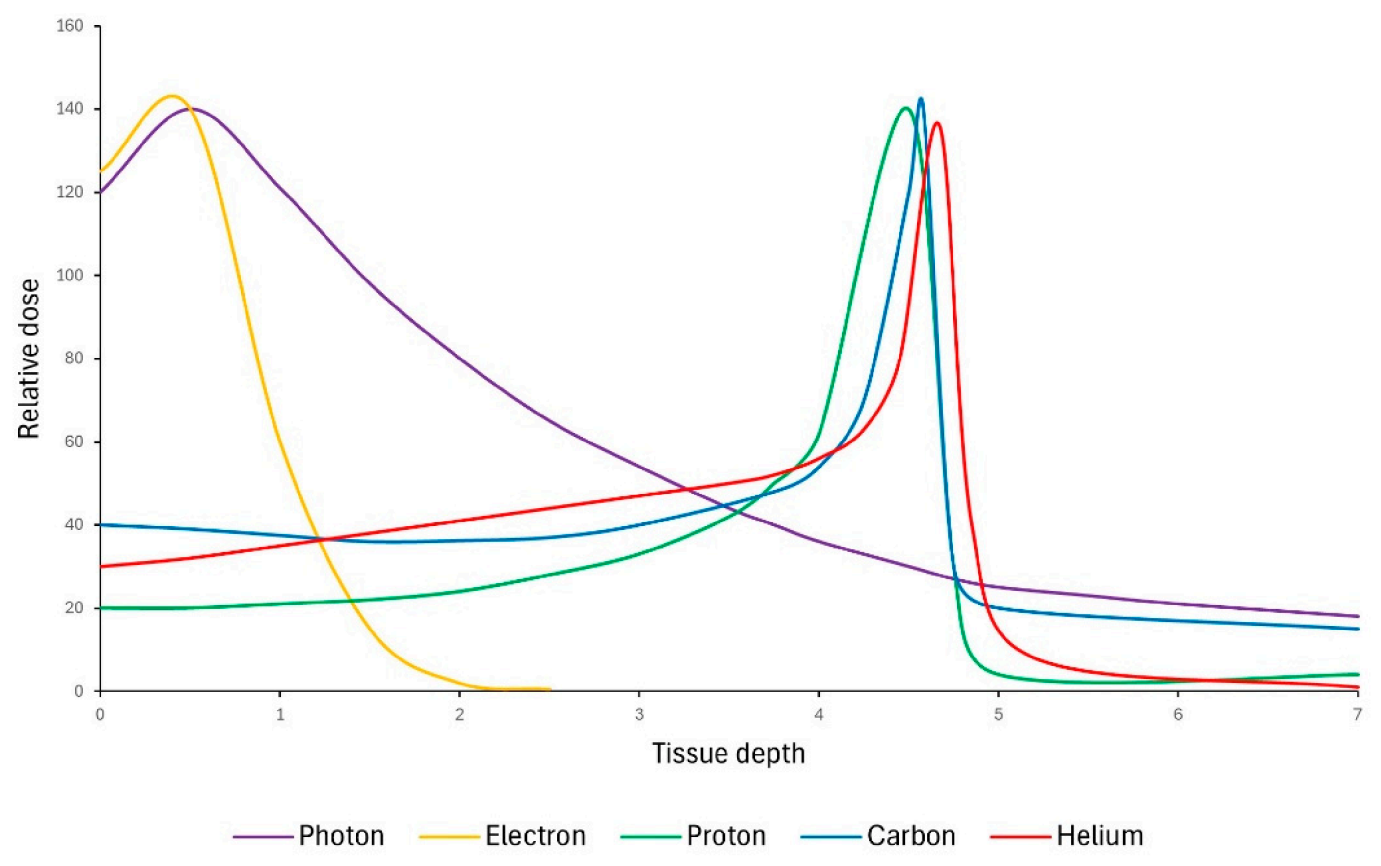
Energy deposition profiles for photons, electrons, protons, carbon and helium ions as a function of depth within tissue. Adapted from [3,14].

**Table 2:** Summary of FLASH versus CONV studies using carbon ions.

Model	Ion Source	Beam Energy (MeV)	LET (keV/μm)	Dose (Gy)	FLASH Dose Rate (Gy/s)	CONV Dose Rate (Gy/s)	Outcome	Reference
CHO-K1 cells	Carbon	280	N/A	7.5	70	0.6	Significant increase in clonogenic survival following FLASH—most pronounced at 0.5% O_2_ No FLASH sparing when irradiated at 21% O_2_	[63]
Human lung fibroblast HFL1 and human salivary gland tumour lines HSGc-C5	Carbon	290	13 (entrance) and 50 (Bragg peak)	1–3	96–195	8–13	FLASH irradiations at the plateau and the SOBP regions of the carbon ion beam showed no sparing effect on growth and senescence of HFL1 cells or for survival of HSGc-C5 cells	[64]
HSGc-c5, Human dermal fibrobast (HDF), and Human lung bronchial epithelial cell line (Nuli-1)	Carbon	N/A	19 (entrance) and 50 (Bragg peak)	2.7, 5.4 or 6.5	100	1.6	FLASH increased survival fractions compared to CONV Higher LET demonstrated greater protective effect γH2AX foci were significantly reduced in FLASH vversus CONV	[65]
B16F10 murine melanoma and Pan02 murine pancreatic adenocarcinoma cell lines	Carbon	N/A	50	5.65–11.1	380	1.16	FLASH dose rates suppressed expression of PD-L1 on Pan02 No difference regarding survival in either cell line across dose rates	[66]
C3H/He mice (posterior limb)—injection with LM8 osteosarcoma cells	Carbon	240	~15	18	~100	0.3	FLASH reduced normal tissue toxicity (muscle structural changes) and tumour growth FLASH reduced number of lung metastases	[67]
C3H/He mice (posterior limb)—injection with LM8 osteosarcoma cells	Carbon (SOBP)	240	~65–85	20	>100	~0.33	FLASH reduced healthy tissue damage (lower collagen deposition indicating reduced fibrosis) Equivalent mild skin toxicity, tumour control and suppression of lung metastases across dose rates No significant effect of FLASH dose rate on local anti-tumour or gut microbiome composition	[68]
C3H/He mice—skin	Carbon (Bragg peak)	290	13	34, 42, 50 or 58	318	0.047–0.051	FLASH-irradiated mice displayed lower skin reaction scores and tissue sparing effect	[69]
C3H/HeSIc mice—intestine	Carbon	400	~11	12–18	~96	~0.3	Survival fractions were similar across dose rates No significant changes in intestinal toxicity	[70]

**Table 3:** Summary of FLASH versus CONV studies using helium ions.

Model	Ion Source	Beam Energy (MeV)	LET (keV/μm)	Dose (Gy)	FLASH Dose Rate (Gy/s)	CONV Dose Rate (Gy/s)	Outcome	Reference
Human lung epithelial cell lines (A549 and H1437) and primary human pulmonary fibroblasts	Helium	145.74	4.5–16	2–12	~185 ± 35	~0.12 ± 0.07	No dose rate dependence of cellular response at 21% O_2_ or following 2 or 4 Gy at 1% O_2_ Greater survival and reduced γH2AX signal at 1% O_2_ for doses ≥ 8 Gy irradiated at a FLASH dose rate	[75]
Zebrafish embryo	Helium	40.3 ± 2.3	27.6	17.9–24.1	9700 ± 820	0.25 ± 0.02	FLASH dose rate spares body development and reduces spinal curvature, relative to CONV dose rate	[76]
C57BL/6 mice—brain and subcutaneous carcinoma tumour model (adenocarcinoma A549 cells)	Helium	145.74	20–40 (brain studies) and 14–37 (tumour model)	10 or 12.5	250	0.2	FLASH reduced brain injury (lower levels of γH2AX and preserved neurovascular endothelium) FLASH resulted in fewer CD68^+^ iNOS+ microglia/macrophages, indicating reduced neuroinflammation Tumour control was similar between dose rates	[77]

## Data Availability

No new data were created or analysed in this study. Data sharing is not applicable to this article.
